# Lead Exposure: A Summary of Global Studies and the Need for New Studies from Saudi Arabia

**DOI:** 10.1155/2014/415160

**Published:** 2014-08-19

**Authors:** A. P. Shaik, S. A. Sultana, A. H. Alsaeed

**Affiliations:** ^1^College of Applied Medical Sciences, King Saud University, Riyadh, Saudi Arabia; ^2^Department of Clinical Laboratory Sciences, College of Applied Medical Sciences, King Saud University, Riyadh, Saudi Arabia

## Abstract

Lead poisoning (plumbism) can cause irreversible genetic and reproductive toxicity, hematological effects, neurological damage, and cardiovascular effects. Despite many efforts to minimize lead poisoning, it continues to be a major health concern in many developing and developed countries. Despite efforts to control lead exposure and toxicity, serious cases of lead poisoning increasingly occur as a result of higher vehicular traffic and industrialization. The biomarkers for identification of genetic susceptibility to a particular disease are useful to identify individuals who are at risk for lead poisoning. Although many such studies have been taken up elsewhere, very few studies were performed in Saudi Arabia to assess susceptibility to lead poisoning. This indicates an urgent need for testing of susceptible individuals. The present paper was planned to understand the genetic susceptibility to lead toxicity in the various population studies conducted worldwide and also to correlate it with the current scenario in Saudi Arabia. Such studies are necessary for appropriate precautions in terms of diet and avoiding exposure to be used in order to prevent adverse health effects.

## 1. Introduction

Genetic biomonitoring of populations exposed to hazardous substances like heavy metals could create an early warning system for genetic diseases [1, 2]. Many types of occupational exposure pose serious health hazards [3]. Lead is a ubiquitous metal that has been used in more than 900 occupations, including battery manufacturing, smelting, and mining [4]. Lead poisoning occurs as a result of ingestion or inhalation of inorganic lead particles or through transdermal absorption of organic alkyl lead [2, 5]. Once absorbed, most of the lead binds to erythrocytes causing most of the toxic effects [6, 7]. Bone lead accounts for more than 95% of the lead burden in adults and 70% of the burden in children [8, 9]. Lead exposure in the general population (including children) occurs primarily through ingestion; however, inhalation also contributes to lead body burden and may be the major contributor for workers in lead-related occupations [10]. Once absorbed, lead may be stored for long periods in mineralizing tissue (i.e., teeth and bones) and then released again into the bloodstream, especially in times of calcium stress (e.g., pregnancy, lactation, and osteoporosis) or calcium deficiency [11].

Saudi Arabia faces many environmental challenges that can impact negatively human health and productivity [12]. The Kingdom of Saudi Arabia is therefore making concerted efforts to monitor and reduce health hazards [13]. Human populations in this region are increasingly becoming affected by heavy metal lead either occupationally (workers in battery manufacturing units, cement manufacturing companies, battery recycling units, and paint manufacturing companies) or nonoccupationally (families living in and near factories and neighbourhood habitations in addition to indirect use of lead in various home remedies such as kohl and bakhoor) [2, 14]. Although leaded gasoline which was once thought to be a major source of lead exposure across different countries has almost been phased out in Saudi Arabia, the multiple other sources as described above can also significantly impact human health.

Unlike overt lead toxicity, where there is usually one identifiable source, low-level environmental exposure to lead is associated with multiple sources including occupational, environmental, and home use [9]. Evaluation of the relative contributions of the different sources is therefore complex and likely to differ between areas and population groups [8]. Classically, lead toxicity has been detected using phenotypic estimations of blood lead level; this is now largely being also supported by genotype analyses of ALAD gene polymorphisms which are known to influence the levels of blood lead and thus directly affect the susceptibility of individuals to lead poisoning. Many such studies have been conducted in different populations. However, the literature and work in this area are very minimal in Saudi Arabia and from the Middle Eastern region further preventing the overall understanding of susceptibility to lead poisoning in this region. Therefore, a systematic review of the literature with focus on genotype frequency analyses conducted in different populations was performed using PubMed (January 1990 to December 2013) to understand where the Kingdom stands in terms of these studies vis-à-vis the published literature. Retrieved articles and their bibliographies were evaluated and reviewed independently by 2 medical experts before the shortlisted studies were further analyzed. Brief descriptions of the clinical implications of lead exposure have also been presented.

## 2. Evaluation of Health Risks and the Pathophysiology of Lead Toxicity

Exposure to lead can have a range of biological effects depending on level and duration of exposure; the developing fetus and infants are more sensitive than adults [15, 16]. Impaired hemoglobin synthesis; kidney dysfunction, gastrointestinal disturbances, and bone and joint defects; and acute or chronic damage to the nervous system are the most reported effects [9, 17]. Reportedly, even intermediate concentrations of lead can have subtle, subclinical effects, particularly on neuropsychological developments in children [6, 9]. In addition to causing a disruption of enzymatic process through interaction with electron-donor groups (-sulfhydryl) [9], lead interferes with the sodium-potassium-adenosine triphosphate (Na^+^/K^+^-ATP) pump and increases cellular fragility [10, 15]. Lead interferes with the biosynthesis of heme at several enzymatic steps; the inhibition of Δ-aminolevulinic acid dehydratase (ALAD) and heme synthetase enzyme and the consequent accumulation of aminolevulinic acid and protoporphyrin [PP] are characteristic of human lead poisoning [18–20] in addition to increased urinary excretion of coproporphyrinogen III [20].

Although ALAD enzyme has a well-established role in heme biosynthesis, only a few studies so far have assessed the effects and extent of lead pollution in the people of Saudi Arabia since 1999 (Table 1; [21–25]). Lead strongly inhibits ALAD enzyme stoichiometrically and at the molecular level displaces a zinc ion at the metal binding site producing inhibition through a change in the enzyme's quaternary structure. Consequently, a build-up of aminolevulinic acid which can be detected in plasma and urine even at blood lead concentrations of less than 10 ug/dL [26–28]. In addition, the accumulated aminolevulinic acid can stimulate *δ*-aminobutyric acid receptors in the nervous system owing to its resemblance to *δ*-aminobutyric acid; this is thought to be one of the primary mechanisms of lead-induced neurotoxicity [29].

## 3. Genetic Susceptibility to Lead Poisoning: ALAD Genotypes

The most commonly studied polymorphism in the ALAD gene, namely, the* ALADG177C,* yields two codominant alleles,* ALAD-1* and* ALAD-2*. The frequencies of ALAD 1-1, ALAD 1-2, and ALAD 2-2 genotypes vary by geography and race [30]. The* ALAD* gene is located on chromosome 9q34 encodes the ALAD enzyme (E.C. 4.2.1.24), also known as porphobilinogen synthase composed of eight identical subunits which requires eight zinc ions as cofactors for full activity [31]. The ALAD-2 allele contains a G → C transversion at position 177 of the coding region, resulting in the substitution of asparagine for lysine at amino acid 59 producing an enzyme which modifies the kinetics of lead upon exposure. ALAD 2-2 and 1-2 heterozygotes produce an enzyme that is more electronegative than that of ALAD-1 homozygotes and thus individuals with these genotypes have increased blood lead levels and lower concentrations of aminolevulinic acid in plasma, lower zinc protoporphyrin levels, lower cortical bone lead concentrations, higher concentrations of trabecular (spongy) bone lead, and lower amounts of DMSA chelatable lead compared to ALAD 1-1 individuals.

## 4. Global Scenario of ALAD Gene Polymorphism Studies

**Figure 1:**
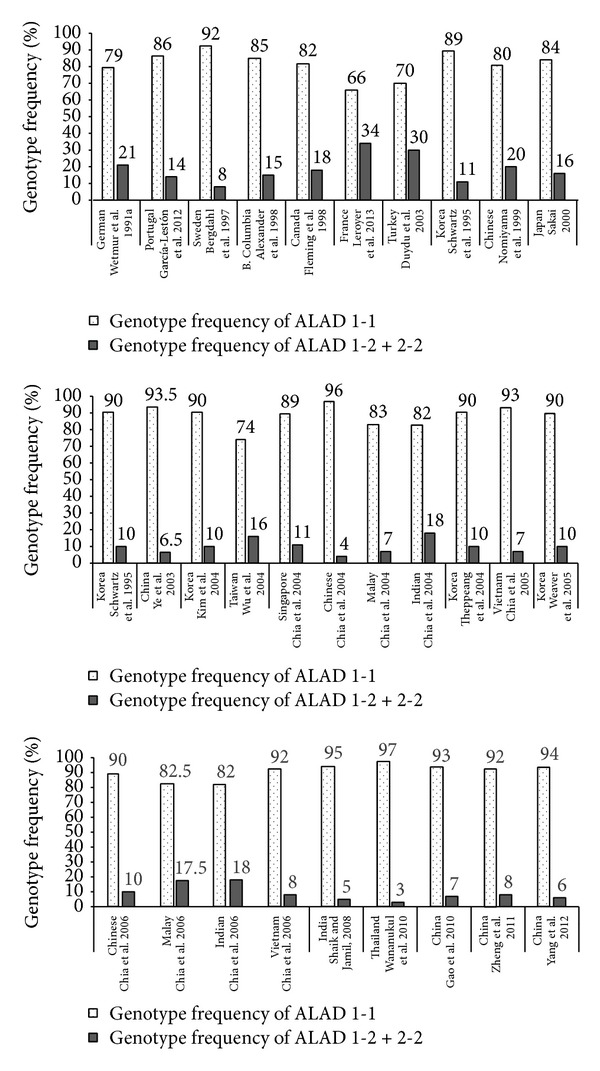
Occupational studies assessing ALAD gene polymorphisms in adults.

**Figure 2:**
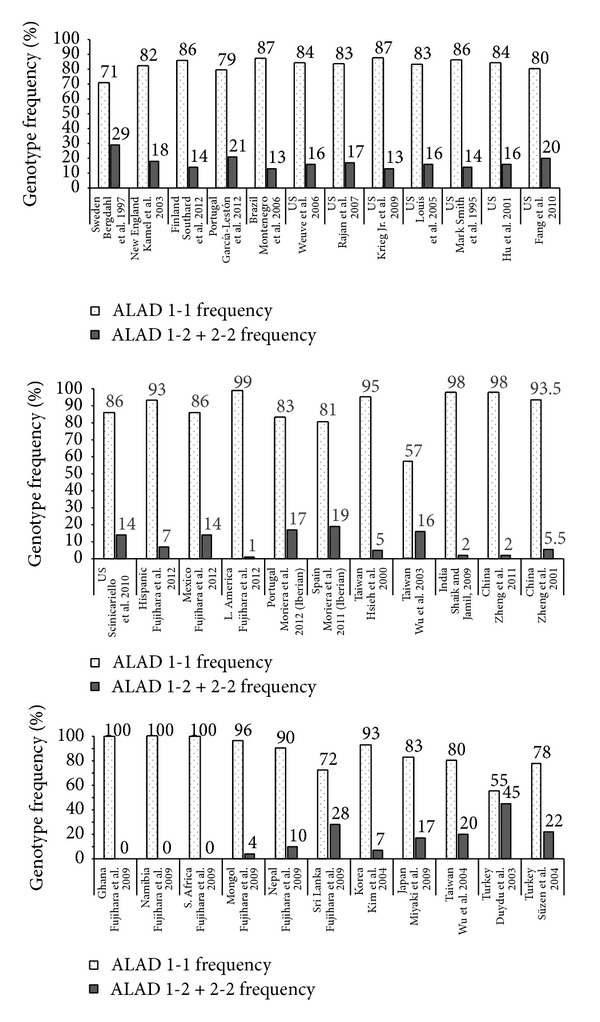
General population and environmental studies assessing ALAD gene polymorphisms in adults.

**Figure 3:**
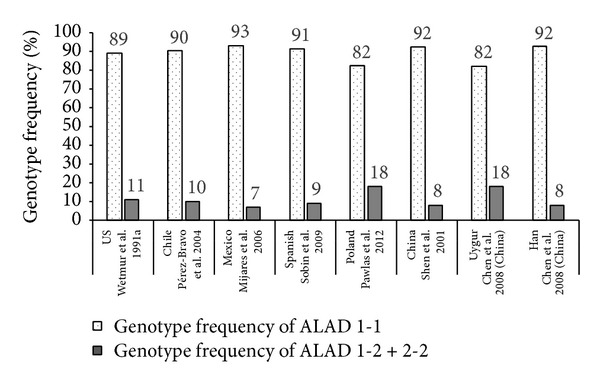
General population and environmental studies assessing ALAD gene polymorphisms in children.

Determining polymorphisms that affect susceptibility to lead poisoning thus allows identification of susceptible groups thus playing an important role in prevention of occupational and nonoccupational health hazards [32]. A number of studies have assessed the role of ALAD gene polymorphisms in lead toxicity globally (Figures 1 and 2) [26, 28, 33–78]. A total of 24 studies conducted in various countries on subjects with occupational exposure to lead have assessed susceptibility to lead toxicity with relation to ALAD gene polymorphisms (Figure 2). Around 26 studies estimated ALAD gene polymorphisms in adults who belonged to general population and who were exposed to environmental lead by virtue of close proximity to lead polluted regions or working in factories but without directly using lead (i.e., administrative roles) (Figure 2). In addition, seven general population and environmental studies estimated ALAD gene polymorphisms in children (Figure 3). The mean blood lead levels in most of the studies conducted on occupational exposure were from 10 to 61 *μ*g/dL. Most of the studies conducted in the general population and in adults and children with environmental exposure showed blood lead levels <10 *μ*g/dL. These studies were predominantly conducted in the Caucasian and Asian populations. Although ALAD 1-2 and 2-2 genotypes were present in lesser numbers, it was evident that subjects with ALAD2 allele showed higher blood lead levels compared to ALAD1 allele. ALAD allele was a significant determinant for blood lead concentrations and it is therefore important to characterize subjects based on their genotype since such information will help in keeping appropriate preventive measures in place especially for subjects who have increased susceptibility to lead poisoning [77].

## 5. Nonoccupational Use of Lead in Home Remedies and Beauty Products in Saudi Arabia

The* Lead Newsletter 2* [14] published in 2008 clearly indicates the use of lead containing folk home remedies such as* al-murrah* in the treatment of colic, stomach aches, and diarrhea;* anzroot* for gastroenteritis;* bint al dahab; bint; bent dahab* for diarrhea, colic, constipation, and general neonatal use;* bokhoor* to calm infants;* cebagin; farouk* for teething powder;* henna* for hair;* kohl and surma* as cosmetic, astringent for eye injuries, umbilical stump, and teething powder; and* satrinj* as teething powder (Table 2).

## 6. Studies from Saudi Arabia

The definition of an elevated concentration of lead in the blood, according to the Centers for Disease Control and Prevention [9], is 10 *μ*g/dL. However, accumulating evidence indicates that some health effects can occur below this threshold. The data from Table 2 shows percentages of lead levels in children and in adults in Saudi Arabia, estimated up to 1999; there are few studies such as these in Saudi Arabia in adults and in adolescents after 1999. One recent study published in 2012 conducted in children from Madinah region [79] showed mean BLL of 5 *μ*g/dL. Breast milk samples from mothers residing near industrial areas showed higher lead levels than from subjects living in areas with no environmental exposure [21]. The study by Yaish et al. [22] in children from Saudi Arabia indicates that lead poisoning is common and poses a major public health hazard and also reported encephalopathy in children with blood lead levels of 50–60 *μ*g/dL. The very few studies so far in Saudi population limit our understanding of the frequency of ALAD genotypes in this region.

Because lead is a commonly used heavy metal, genetic testing to identify subjects who are predisposed to react adversely to this particular environmental exposure could play a significant role in efforts to reduce the disease burden associated with lead toxicity [8]. The Saudi Arabian population is comprised of a unique genetic makeup and it is therefore important to study the Arab populations to understand the extent of ALAD gene variations and to understand if this population is less or more prone to lead poisoning. Given the heterogeneity of the Arab populations, and the absence of such studies in this region, ALAD genetic polymorphism studies will help in raising awareness to lead toxicity.

Although physicians have a very good understanding of the therapeutic measures that need to be taken in case of toxicity, it is essential for research scientists to work towards bridging the gap between understanding the toxic effects of exposure and their modulations at a cellular and molecular level in order to devise appropriate treatment regimen. Genetic monitoring studies in the Kingdom of Saudi Arabia that employ a holistic approach towards understanding lead toxicity will help physicians to concentrate more on individualistic therapies. Identification of susceptibility through population-based screening methods will definitely improve understanding of the health effects of heavy metal lead in the Saudi Arabian population. This approach will aid in appropriate management of risk factors and mitigating lead poisoning through community-wide awareness programs.

## Figures and Tables

**Table 1:** Use of heavy metal lead in traditional medicine in Saudi Arabia.

Exposure source	Description/exposure pathway	Ref. #
Bint dahab	A yellow lead oxide used as a home remedy.	McNiel and Reinhard 1967 [80]
Santrinj	98% lead oxide used as a home remedy for “gum boils” and “teething.”	McNiel and Reinhard 1967 [80]
Traditional Saudi medicine	Orange powder for teething and for antidiarrheal effect.	Abu Melha et al. 1987 [81]
Kohl	83% lead, believed to strengthen and protect the eyes.	Al-Saleh et al. 1999 [23]

**Table 2:** Studies that estimated lead levels in Saudi Arabia.

References	Lead levels	Percentage of subjects
Al-Saleh 1995 [24]	5–10 *μ*g/dL	23.3
Al-Saleh et al. 1995 [25]	10–20 *μ*g/dL	15.7
Younes et al. 1995 [21]	0.318–2.5 *μ*g/dL	81% of nursing mothers from environmental lead exposure
Al-Saleh et al. 1999 [23]	>20 *μ*g/dL	11.4
